# Supplementary material for: Effects of Arbuscular Mycorrhization on Fruit Quality in Industrialized Tomato Production
Source: Int J Mol Sci. 2020 Sep 24;21(19):7029. doi: 10.3390/ijms21197029 (PMC7582891; doi:10.3390/ijms21197029)
Supplement: Supplementary file 1 [file ijms-21-07029-s001.zip › Figure S2.pdf]

Supplemental Figure S2

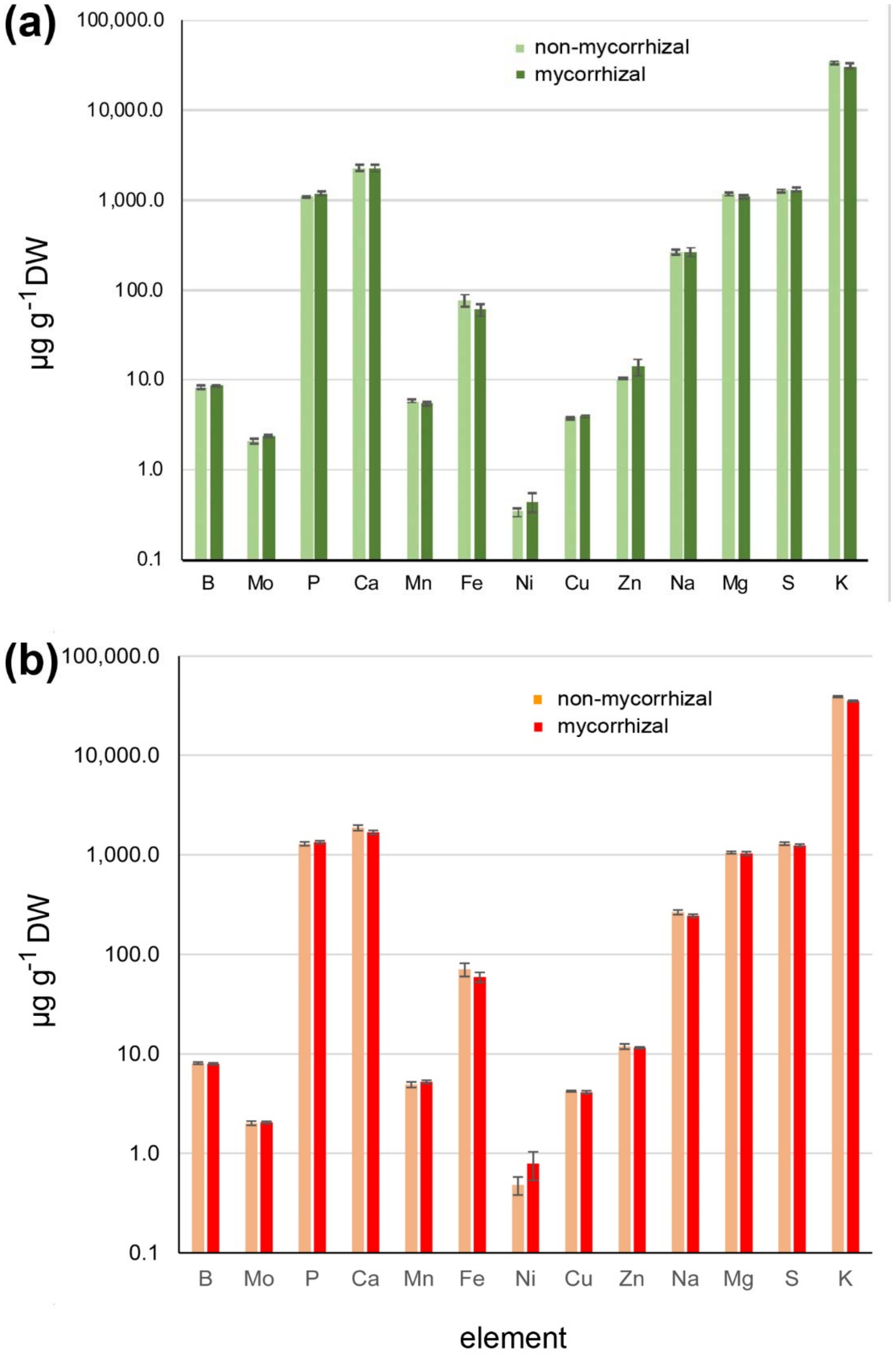

Fig. S2: Content of minerals in green (a) and red (b) fruits from non-mycorrhizal and mycorrhizal plants. Note that there is no difference between levels of all minerals determined. Values are given as mean ± SE (n = 8).
