# Supplementary material for: Effects of Arbuscular Mycorrhization on Fruit Quality in Industrialized Tomato Production
Source: Int J Mol Sci. 2020 Sep 24;21(19):7029. doi: 10.3390/ijms21197029 (PMC7582891; doi:10.3390/ijms21197029)
Supplement: Supplementary file 1 [file ijms-21-07029-s001.zip › Figure S1.pdf]

Supplemental Figure S1

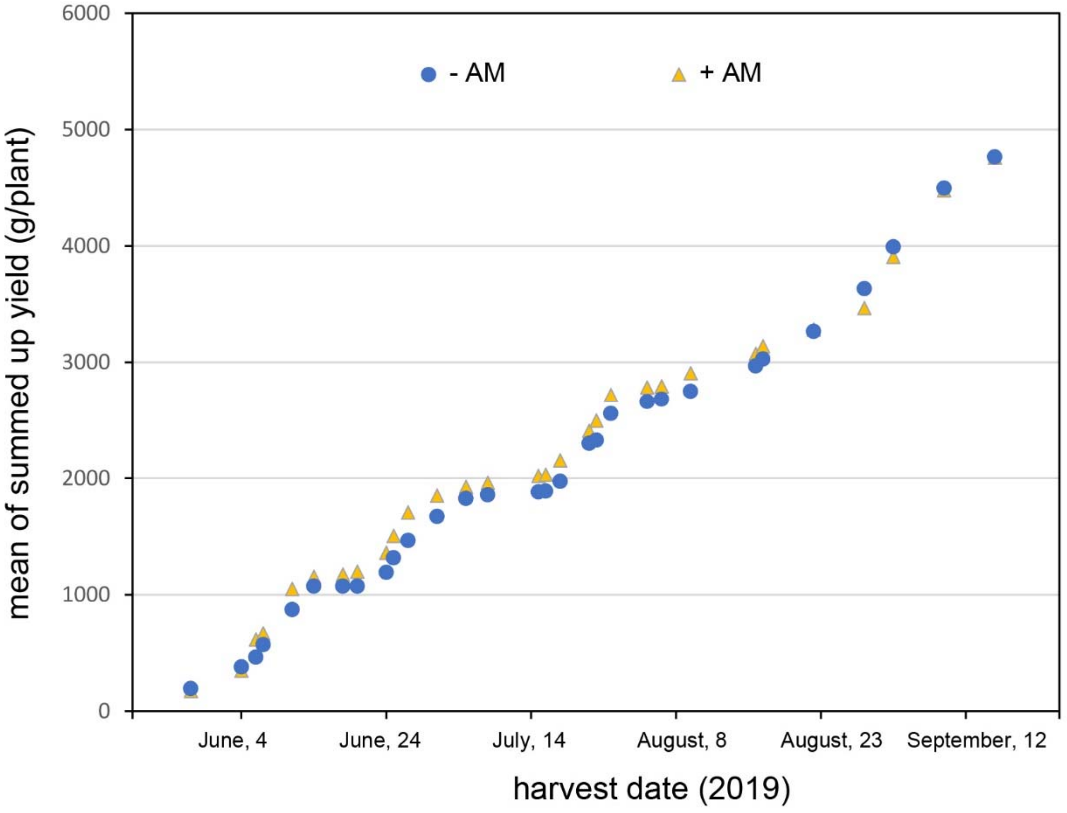

Fig. S1: Yield of tomato plants cv. Picolino/Maxifour grown under commercial conditions in greenhouse at INOQ GmbH, 2019. Note that there is no difference between yields from non-mycorrhizal (- AM) and mycorrhizal (+ AM) plants.
